# Persistently elevated β-human chorionic gonadotropin level after vacuum-assisted uterine aspiration: a case report

**DOI:** 10.1186/s13256-022-03511-7

**Published:** 2022-07-17

**Authors:** Annika Sinha, Betsy Patterson

**Affiliations:** Department of Obstetrics and Gynecology, Cleveland Clinic Foundation, 9500 Euclid Ave, Cleveland, OH 44195 USA

**Keywords:** Urothelial carcinoma, Case report, Beta-human chorionic gonadotropin hormone, Spontaneous abortion

## Abstract

**Background:**

Urothelial carcinoma of the bladder accounts for nearly 90% of all bladder cancers. Risk factors include cigarette smoke, chronic cystitis, and human papilloma virus infection. It is commonly diagnosed by hematuria, obstructive voiding, and irritative symptoms. Despite the prevalence of urothelial carcinoma, elevation of β-human chorionic gonadotropin in the setting of these malignancies is not common. This case report informs gynecologic practitioners to consider urologic causes of β-human chorionic gonadotropin elevation even in the setting of recent spontaneous abortion and details comprehensive review of diagnostic testing in the setting of β-human chorionic gonadotropin elevation.

**Case presentation:**

A 49-year-old, non-Hispanic Caucasian woman, former smoker, with a history of polycystic ovary syndrome, high-risk human chorionic gonadotropin infection, and hypertension, underwent vacuum-assisted aspiration for significant vaginal bleeding in the setting of incomplete abortion. Fetal tissue was confirmed pathologically. Human chorionic gonadotropin levels decreased to 12.5 mU/mL and were no longer followed due to resumption of menses. Five months later during routine preoperative evaluation for orthopedic surgery, her human chorionic gonadotropin level was found to be elevated. She was also noted to have persistent asymptomatic hematuria. She completed an extensive gynecologic and urologic work-up, including hysteroscopy, dilation and curettage, methotrexate therapy, computerized tomographic imaging, and cystoscopy to finally arrive at the diagnosis of urothelial carcinoma.

**Conclusions:**

Only a rare subset of urothelial carcinomas secretes β-human chorionic gonadotropin. Therefore, diagnosis of urothelial carcinoma is typically achieved by urine cytology with cystoscopic biopsy. Although rare, urothelial carcinoma should be considered in patients with risk factors presenting with persistently elevated beta-human chorionic gonadotropin.

## Background

Urothelial carcinoma (previously transitional cell carcinoma) of the bladder is the ninth most common cancer and usually presents between the ages of 50 and 60 years in adult patients [1]. Risk factors for this cancer include cigarette smoking, occupational carcinogen exposure, excessive chlorine exposure, chronic cystitis, human papilloma virus (HPV) infection, poor fluid intake, certain medications, high arsenic load, hereditary cancer syndromes, and aristolochic acid use [1]. Presenting symptoms include hematuria, irritative symptoms, or obstructive voiding. Diagnosis is typically achieved by urine cytology with cystoscopic biopsy. Case report data suggest that serum β-human chorionic gonadotropin (HCG) can be produced by urothelial carcinoma [1–3]. Despite this known phenomenon, diagnosis of urothelial carcinoma by HCG elevation is rare, and persistently elevated HCG levels may warrant not only a gynecologic, but also urologic work-up.

## Case

The following is a case of persistently elevated β-HCG levels five months after vacuum-assisted aspiration of an incomplete abortion. A 49-year-old non-Hispanic, Caucasian woman, gravida 1 para 0, with polycystic ovarian syndrome marked by irregular periods, former smoking history, high-risk HPV infection, and hypertension, presented to the emergency department with heavy vaginal bleeding and passage of fetal tissue in the setting of newly diagnosed pregnancy. On admission to the emergency department, she was normothermic and normotensive with a normal heart rate. Her physical examination revealed significant vaginal bleeding, and bedside ultrasonography revealed a slightly heterogeneous endometrial lining. Manual vacuum aspiration was completed at the bedside for incomplete abortion. Analysis of her urine showed significant hemoglobin, which was attributed to her vaginal bleeding. Surgical pathology confirmed 12.5-grams fetal tissue and 20-grams placenta, consistent with 11-week gestational age. On admission to the emergency department, HCG level was 1800 mU/mL, which trended downward to 12.5 mU/mL within 17 days (Table 1). Additional HCG level was not pursued to establish a return to normal HCG level due to regular menses and patient adherence to follow-up. Two months after her initial emergency department presentation, she saw Obstetrics and Gynecology (OB-GYN) to complete routine Pap testing, which showed no epithelial abnormality, but she tested positive for other high-risk HPV subtypes, and was planned to follow routine cervical cancer screening guidelines.

**Table 1:** HCG trend through the clinical course

Date of collection	Laboratory test	Value	Relevant clinical history
7 April 2020	HCG quantitative	1800 mU/mL	Presentation for incomplete abortion
24 April 2020	HCG quantitative	12.5 mU/mL	Post-abortion surveillance
2 September 2020	HCG quantitative	23.6 mU/mL	Presentation for non-gynecologic, orthopedic surgery
9 September 2020	HCG quantitative	20.8 mU/mL	Repeat testing for verification of HCG result
11 September 2020	HCG quantitative	24.5 mU/mL	Evaluation of hypothalamic-pituitary axis
	FSH	10.7 mU/mL	
	LH	5.0 mU/mL	
	HCG qualitative	Positive	
19 October 2020	HCG quantitative	22.6 mU/mL	Evaluation of HCG level after hysteroscopy
22 October 2020	HCG quantitative	28.7 mU/mL	Pre-methotrexate therapy
22 October 2020	HCG quantitative	27.0 mU/mL	Post-methotrexate therapy
4 November 2020	HCG quantitative	27 mU/mL	Prior to urologic resection of bladder tumor
9 December 2020	HCG quantitative	< 1 mU/mL	Post-urologic resection of bladder tumor

Human chorionic gonadotropin (HCG) reference range < 5.0 mU/mL. Follicle-stimulating hormone (FSH) reference range based on phase—follicular, 2–11 mU/mL; mid-cycle, 10–30 mU/mL; luteal, 1–9 mU/mL; postmenopausal, 20–100 mU/mL. Luteinizing hormone (LH) reference range based on phase—follicular, 1–12 mU/mL; mid-cycle, 20–90 mU/mL; luteal, 1–10 mU/mL; postmenopausal, > 20 mU/mL

Five months after her vacuum-assisted aspiration, the patient was scheduled to complete orthopedic surgery. However, her urine HCG testing was positive on the day of surgery. Patient was asymptomatic, and the quantitative HCG level was 23.6 mU/mL on the day of surgery. Patient was referred to OB-GYN for management of HCG findings. One week later, repeat HCG testing showed an HCG quantitative level of 20.8 mU/mL, and nine days after her planned orthopedic surgery date, HCG quantitative level was 24.5 mU/mL, and HCG qualitative urine testing was still positive. To rule out pituitary HCG production, follicle-stimulating hormone (FSH) levels and luteinizing hormone (LH) levels were drawn and found to be normal.

One month after diagnosis of persistent HCG levels, saline-infusion sonography was completed due to concern of possible retained products of conception. It showed two discrete calcifications in the endometrium, and hysteroscopic evaluation was recommended. At her preoperative evaluation for her planned hysteroscopy, she complained of urinary odor for which another analysis of her urine was sent, showing hemoglobin (Table 2). Therefore, the patient requested referral to urology. Hysteroscopy with dilation and curettage, and hysteroscopic myomectomy was completed. Surgical pathology showed benign fibroid with no evidence of retained products of conception or neoplastic tissue.

**Table 2:** Findings on urine analysis during clinical course

Date	Hemoglobin	Red blood cell count	Nitrites	Leukesterase	Relevant clinical history
7 April 2020	Large	Many	Negative	Trace	Presentation for incomplete abortion
19 October 2020	Large	N/A	Negative	Negative	After hysteroscopy
1 December 2020	2+	11–25	Negative	25	Post-tumor biopsy
25 March 2021	Trace	N/A	Negative	Trace	Post-tumor resection on BCG treatments
6 April 2021	Trace	11–25	Negative	Negative	Pre-cystoscopic surveillance after BCG treatment round 1
28 July 2021	Negative	N/A	Negative	Negative	Pre-cystoscopic surveillance after BCG treatment round 2
12 October 2021	Negative	N/A	Negative	Negative	Post-pelvic exenteration

Hemoglobin reference: negative; red blood cell count reference: 0–3 cells per high-power field; nitrite reference: negative; leukesterase reference: negative

Despite her hysteroscopic procedure, post-procedure serum HCG quantitative level was 22.6 mU/mL one week later. Therefore, a decision was made to complete methotrexate therapy for concern for possible ectopic pregnancy. One week after methotrexate therapy, HCG level was still 24 mU/mL. Therefore, Gynecology Oncology was consulted, and the patient was recommended to complete a more sensitive test for β-HCG, named “beta sub-unit HCG tumor marker.” Now, two months after her orthopedic surgery date, this highly sensitive β-HCG quantitative tumor marker level was again elevated at 27 mU/mL.

Owing to her request, the patient saw Urology for hematuria, who recommended computerized tomography (CT) urogram, which was significant for microlobulated enhancing bladder mass within superior anterior urinary bladder with some punctate faint wall calcifications. Urology recommended cystoscopy and transurethral bladder tumor resection, and Gynecology Oncology recommended repeat β-HCG tumor marker level in three weeks, oral contraception for ovarian suppression, and follow-up on bladder surgical pathology. Surgical pathology from her cystoscopic resection was consistent with high-grade papillary urothelial carcinoma with invasion into the lamina propria. Macroscopically, the specimen was described as irregular fragments of soft tan-brown tissue that measure 3.5 × 1.9 × 0.3 cm with an aggregate weight of 1.0 grams. Post-procedure β-HCG quantitative tumor marker level was 5 mU/mL and, one month after tumor removal, β-HCG quantitative tumor marker level < 1 mU/mL. The patient completed urologic restaging and completed immunotherapy instillations with Bacillus Calmette–Guérin (BCG) with two rounds of induction; however, she was noted to have persistent urothelial carcinoma *in situ* and severe dysplasia on two subsequent post-BCG treatment cystoscopic biopsies four months and seven months after initiation of BCG. Therefore, given high risk of recurrence, she underwent complete anterior pelvic exenteration with ileal conduit one year after diagnosis of persistently elevated HCG levels. She currently has no evidence of disease.

## Conclusion

Persistently elevated HCG levels require diagnostic evaluation with careful consideration of the following etiologies, including ectopic pregnancy, endocrinopathy, retained products of conception after uterine evacuation, and neoplastic etiologies. Any elevation of HCG without documented intrauterine pregnancy should be considered pathologic and be investigated as such. As in this case, a thorough evaluation of the hypothalamic-pituitary axis, uterine cavity, and urinary system should be completed. Particularly, when considering neoplastic work-up, both gynecologic and urologic carcinomas should be considered. More commonly, β-HCG elevations are related to gynecologic malignancies, such as trophoblastic disorders, and ovarian neoplasms. However, case report data suggest that β-HCG elevation can be found in urothelial carcinoma.

Although urothelial carcinoma is a common bladder malignancy, diagnosis is rarely elucidated with β-HCG elevation, which is a limitation of the application of this finding for diagnosis of urothelial carcinoma in other populations. Use of tissue, serum, or urine biomarkers for diagnosis of urothelial carcinoma is controversial as it is characterized as a heterogeneous disease without one set of distinct tumor markers [4]. Currently, biomarkers, such as nuclear matrix protein 22, bladder tumor antigen (BTA), and BTA tak, ImmunoCyt, UBC test, Cyfra 21-1, and Cxbladder monitor, have been studied for diagnosis [4]. Notably, β-HCG is not a routine or recommended screening biomarker for urothelial cancer. In this case, HCG was also not used as a marker of monitoring treatment, which is another limitation to the use of HCG in the setting of urothelial carcinoma. Instead, surveillance through cystoscopic bladder biopsies is recommended for treatment monitoring after diagnosis. However, this patient demonstrated multiple risk factors for urothelial carcinoma, including high-risk HPV-positive status, former smoking, and hematuria, warranting further urologic evaluation. On discussion with the patient on her perspective, she is grateful for the dedicated gynecologic and urologic care and recommends advocating for abnormal urine analysis results, as she demonstrated when she requested urologic consultation, which led to her final diagnosis.

## Data Availability

The datasets used and/or analyzed during the current study are available from the corresponding author on reasonable request.

## Abbreviations

HPV: Human papilloma virus
HCG: Human chorionic gonadotropin
OB-GYN: Obstetrics and Gynecology
FSH: Follicle-stimulating hormone
LH: Luteinizing hormone
CT: Computerized tomography
BCG: Bacillus Calmette–Guérin
BTA: Bladder tumor antigen
